# Clinical Characteristics and Outcomes of Patients with Anti-MDA5 Antibody Associated Rapidly Progressive Interstitial Lung Disease (RP-ILD): A Case Series

**DOI:** 10.31138/mjr.020324.cco

**Published:** 2024-12-31

**Authors:** Harikrishnan Gangadharan, Anusree Prasad Seetha, Sajitha Musthafa, Padmanabha Shenoy, Maria Francis, Arjun Krishna, Vaishnavi Kamath, Venugopal KP, Radha Kumar

**Affiliations:** 1 Department of Rheumatology, Government Medical College Kottayam, Kerala, India,; 2 Department of Pulmonary Medicine, Government Medical College Kottayam, Kerala, India,; 3 Centre for Arthritis and Rheumatism Excellence (CARE), Cochin, Kerala, India,; 4 Holy Ghost Hospital Muttuchira, Kottayam, Kerala, India,; 5 Department of Internal Medicine, Government Medical College Kottayam, Kerala, India

**Keywords:** rapidly progressive interstitial lung disease, inflammatory myopathy, anti-MDA5 antibody

## Abstract

**Objective:**

To describe the clinical profile and treatment outcomes of a longitudinal series of patients with rapidly progressive interstitial lung disease (RP-ILD) associated with anti MDA 5 antibody.

**Methods:**

RP-ILD patients were identified from a prospective cohort of adult patients with idiopathic inflammatory myopathy (IIM). Clinical, demographic, and serological parameters of all patients were recorded using a structured proforma. Rapidly progressive ILD was defined as the development of radiological deterioration and hypoxemia within 3 months of the onset of respiratory symptoms. The diagnosis of RP-ILD was made after high-resolution CT chest and multidisciplinary discussion. RPILD patients were followed up with serial pulmonary function tests (PFT) every 3 months and echocardiography every 6 months.

**Results:**

Among 58 patients with IIM, five patients (3 female, 2 male) had RP-ILD. All the five patients had amyopathic presentation with polyarthritis, negative anti-nuclear antibody (ANA) and strong positivity (3+) for anti MDA 5 antibody by line immunoblot assay. The patients were treated with various combinations of immunosuppressants/immunomodulators. Two patients expired, one had stabilisation of lung function and the other two patients showed improvement of lung function over a median follow up of 24 months. High levels of serum ferritin and LDH were seen in non-survivors.

**Conclusion:**

A clinically amyopathic presentation with polyarthritis, negative ANA and a favourable long-term response to combination immunosuppressive therapy defined the clinico-serological profile and treatment response of our anti MDA5 positive RP-ILD patients.

## INTRODUCTION

Anti-melanoma differentiation-associated gene 5 (anti-MDA5) is a myositis-specific autoantibody seen in 10–35% of patients with dermatomyositis.^1^ The clinical spectrum of Anti-MDA 5 associated dermatomyositis is diverse and depends on the ethnicity of the study population. In the Caucasian population, the presentation is milder with arthritis and skin involvement whereas in the Asian population severe clinical features like rapidly progressive interstitial lung disease (RP-ILD) with minimal muscle weakness are more common.^2^ Rapidly progressive ILD is defined as the development of radiological deterioration and hypoxemia within 3 months of the onset of respiratory symptoms.^3^ The mortality associated with RP-ILD is high and often a combination immunosuppressive regime is required for disease control.^4^ We aim to describe the clinical profile and treatment outcome of a longitudinal series of patients who presented with RP-ILD secondary to anti-MDA-5 antibody. We believe our case series can add to the scant literature from the Indian subcontinent on the clinical features and treatment outcomes of RPILD associated with anti-MDA-5 antibody.

## MATERIALS AND METHODS

Out of a prospective cohort of 58 consecutive adult patients who fulfilled the 2017 EULAR/ACR classification criteria of idiopathic inflammatory myopathies (IIM)/Sontheimer classification criteria for amyopathic dermatomyositis^5,6^ and attended the Rheumatology clinic/admitted in wards of a tertiary care hospital in South India between October 2020 and December 2023, 7 patients tested positive for anti MDA 5 antibody. Among these 7 patients, 5 patients developed rapidly progressive interstitial lung disease (RP-ILD). Clinical, demographic, and serological parameters of all patients were recorded using a structured proforma. Myositis-specific autoantibodies (MSA) and myositis-associated autoantibodies (MAA) to 16 antigens were tested by line blot assay using Euroimmun kit (Luebec, Germany) in all patients. Results were categorised as 0–5 (neg), 6–10 (borderline), 11–25 (+) and 26–50(++), strong positive (+++) respectively. Titres of 2+ and above (semiquantitative values of 25 and above) were taken as positive. Anti-Nuclear Antibody (ANA) was tested by immunofluorescence assay (IFA) using Hep-2010 cell line at a dilution of 1:100. Dyspnoea was graded according to the modified medical research council (mMRC) dyspnoea scale.7 Rapidly progressive ILD was defined as the development of radiological deterioration and hypoxaemia within 3 months of the onset of respiratory symptoms. The diagnosis of RP-ILD was made after high-resolution CT chest and multidisciplinary discussion. RP-ILD patients were followed up with serial pulmonary function tests (PFT) every 3 months and echocardiography every 6 months. Written informed consent was obtained from all the participants and the study was approved by the Institutional Review Board (IRB No: 290/2023).

## CASE DESCRIPTIONS

### Case 1

**Figure 1.**
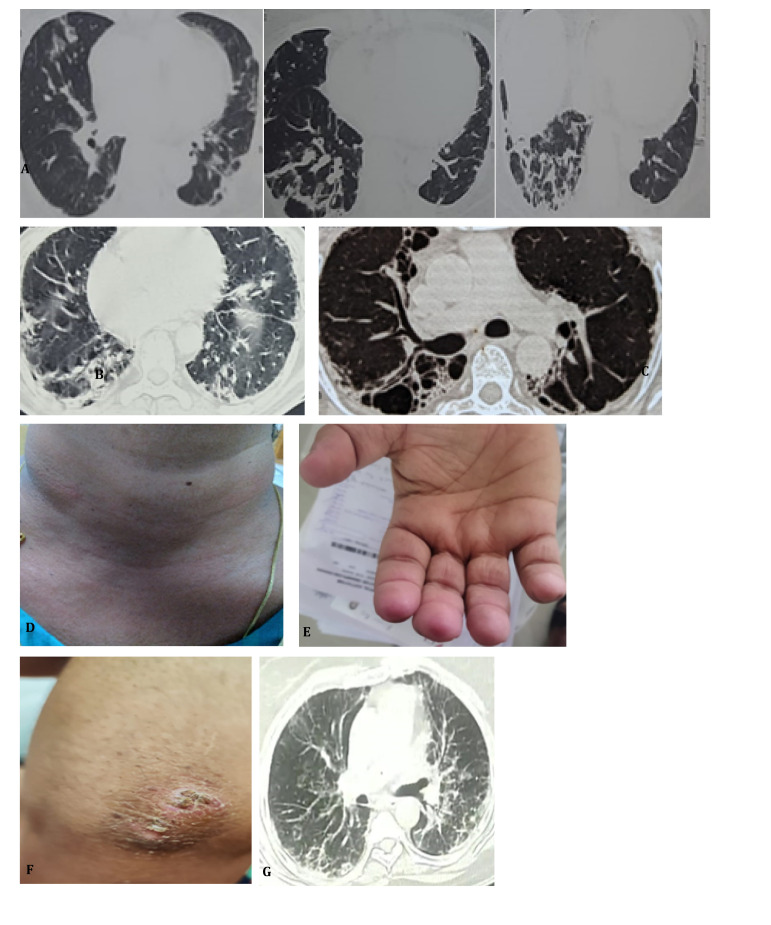
Clinical and radiological images of patients with anti-MDA 5 related RP-ILD. **(A)** High resolution CT chest axial images of case 1 showing bilateral intralobular septal thickening and traction bronchiolectasis and bronchiectasis with basal predominance. **(B)** High resolution CT chest axial images of case 2 showing ground glass opacities and dense septal inflammation with traction bronchiolectasis which progressed to fibrosis and nonspecific reparative cysts and traction bronchiectasis **(C)**. Erythematous rash on anterior aspect of neck suggestive of V sign in Case 3 **(D)** and palmar papules at the tip of index and middle finger of right hand in Case 4 **(E)**. Gottron’s papules over the extensor side of right elbow **(F)** and high-resolution CT chest axial images showing bilateral interstitial infiltrates, ground glass opacities and septal thickening in bilateral lower lobes in Case 5 **(G)**.

A 58-year-old lady presented with inflammatory polyarthritis, Raynaud’s phenomenon, sicca symptoms, and recurrent oral ulcers for the past 8 months. She developed dyspnoea which progressed from mMRC grade 1 to grade 3 over 2 months before admission at our centre. Upon examination, her manual muscle testing (MMT8) score was 80/80. High-resolution CT (HRCT) Chest showed multifocal ground glass opacities with interstitial thickening in bilateral lung fields, predominantly in the lower lobes (**Figure 1A**). Given her RP-ILD and amyopathic presentation, anti-MDA 5 associated RP-ILD was suspected. Myositis autoantibody panel showed 3+ positivity for anti MDA5 antibody. She was treated with intravenous methylprednisolone 1 gram for 3 days, intravenous cyclophosphamide (15mg/kg dose), and oral tacrolimus was started at 2mg per day. However, her respiratory status deteriorated, and she required invasive ventilation. Endotracheal aspirate cultures did not show any evidence of infections and considering the refractory ILD, she was initiated on plasmapheresis. Though she had mild improvement in hypoxemia after the third session of plasmapheresis, she succumbed to cardiac arrest on day 14 of admission.

### Case 2

A 46-year-old male presented with inflammatory polyarthritis and dyspnoea on exertion for five months duration. Three months before admission at our centre, he was evaluated at an outside centre and diagnosed with idiopathic Non-specific interstitial pneumonia and started on oral corticosteroids and azathioprine. Over the next 2 months, his dyspnoea progressed from MMRC grade 2 to grade 4 and it was at this point he came to our centre. Upon examination, he was hypoxemic and required oxygen support. HRCT Chest was suggestive of RP-ILD (**Figure 1B–C**). Myositis autoantibody panel showed anti-MDA 5 +++ (**Table 1**). He was treated with intravenous pulse methylprednisolone 1 gram infusion for 3 days, 6 doses of monthly cyclophosphamide 1 gram each (15mg/kg), and oral cyclosporine 400 mg/day. He required domiciliary oxygen therapy for 6 months after discharge from our centre and has been off oxygen support for the last 34 months. He has completed 40 months of follow-up at our centre and serial pulmonary function tests have shown improvement.

**Table 1.** Summary of clinical profile, therapeutic agents used, and outcomes of 5 cases of RP-ILD in anti- MDA5 positive Dermatomyositis.

	**Case 1**	**Case 2**	**Case 3**	**Case 4**	**Case 5**
**Age**	**58**	**48**	**47**	**32**	**46**
**Gender**	**F**	**M**	**F**	**M**	**F**
**Duration of symptoms (months)**	8	12	5	7	2
**Onset of dyspnoea to development of hypoxemia (months)**	2	2	3	1	0.5
**Duration of follow up after diagnosis of RP-ILD (months)**	Nil Expired on Day 14 after diagnosis of RP-ILD	40	20	24	Nil Expired on Day 4 after diagnosis of RP-ILD
**Extra pulmonary features**	Polyarthritis, Raynaud’s, Sicca symptoms, Recurrent oral ulcer	Polyarthritis	Fever, Polyarthritis Rash Oral ulcers Alopecia Heliotrope sign, V sign, shawl sign, Gottron’s papule	Proximal muscle pain, Polyarthritis Rash Heliotrope sign, Shawl sign, V sign, palmar papules	Fever Polyarthritis Rash Gottron’s Papules V sign
**MMT8 score (out of 80)**	80/80	80/80	80/80	80/80	80/80
**CK (U/L) (35–190)**	114	52	43	68	77
**LDH (U/L) (230–460)**	1060	238	268	334	666
**Ferritin (ng/ml) (10–290)**	1500	N.A	242	N.A	512
**ANA IF**	negative	negative	negative	negative	negative
**MSA/MAA**	Anti-MDA 5+++	Anti-MDA 5+++	Anti-MDA 5+++ Anti Ro 52+++	Anti-MDA 5+++	Anti-MDA 5+++
**PFT**	N.A	Baseline: Not able to perform Last visit: FVC-2.25 L/min (59%) FEV1-1.88 L/min (61%)	Baseline: FVC-1.72 L/min (64%) FEV1- 1.62 L/min (66%) Last visit: FVC- 1.68 L/min (61%) FEV1- 1.56 L/min (63%)	Baseline: not able to perform Last visit: FVC 2.06 L/min (44%) FEV1- 1.92 L/min (40%)	Baseline FVC-1.60 L/min (76%) FEV1-1.32 L/min (74%)
**HRCT Chest**	Multifocal GGOs with septal thickening in bilateral lower lobes	Bilateral basal and subpleural areas of honeycombing, cysts and reticulations	Interstitial thickening, traction bronchiectasis predominantly in both lower lobes	Bilateral interstitial thickening, reticulations, basal predominant multifocal areas of fibrosis	Bilateral interstitial infiltrates, GGOs and septal thickening in lower lobes and upper lobes COVID 19 RT
**Infection work-up**	COVID 19 RT PCR negative CMV DNA PCR-negative Endotracheal aspirate Bacterial, fungal, mycobacterial cultures sterile	COVID 19 RT PCR negative CMV DNA PCR negative	COVID 19 RT PCR negative BAL bacterial culture- no growth BAL fungal stain negative BAL AFB stain negative	COVID 19 RT PCR- negative Sputum Bacterial culture- sterile Sputum AFB stain-negative	PCR- negative Endotracheal aspirate Bacterial, fungal, mycobacterial cultures- sterile CMV DNA PCR negative
**Treatment**	iv methylprednisolone 1 g x 3 doses Iv cyclophosphamide 750mg- 1 dose Tacrolimus 2mg/day Plasmapheresis- 3 sessions	iv methylprednisolone 1 g x 3 doses Iv cyclophospha- mide 1000mg- 6 doses Oral cyclosporine 400 mg/day	iv methylprednisolone 1 g x 3 doses tofacitinib 5mg twice a day	oral prednisolone 1mg/kg, mycophenolate mofetil 2g/day, tacrolimus 4mg/day	iv methylprednisolone 750 mg x 3 doses, IVIG
**Outcome**	Death (day 14 of admission)	Alive Improvement of lung function	Alive Stabilisation of lung function	Alive Improvement of lung function	Death (day 4 of admission)

MMT8: manual muscle testing 8; CK: creatinine kinase; LDH: Lactate dehydrogenase; U/L: Units per litre; ng/ml: nanogram per ml; ANA: anti-nuclear antibody; IF: immunofluorescence; MSA: myositis specific autoantibody; MAA: myositis associated autoantibody; BAL: bronchoalveolar lavage; PFT: pulmonary function test; FVC: forced vital capacity; FEV1: forced expiratory volume in 1 second; N.A: not available; HRCT: High: resolution computed tomography; GGO: ground glass opacity; CMV: cytomegalovirus.

### Case 3

A 47-year-old lady presented to us with photosensitive rash, fever, polyarthritis, alopecia, and oral ulcers for 5 months. Two months after the onset of symptoms, she was treated elsewhere with mycophenolate mofetil and oral prednisolone 50 mg/day as her myositis autoantibody panel showed anti-MDA 5 +++ and Anti Ro 52 +++. When she presented to our centre, she had heliotrope rash, shawl sign, V sign (**Figure 1D**), and Gottron’s papules. Respiratory system examination was normal and MMT 8 score was 80/80. She was continued on mycophenolate and oral steroids were slowly tapered. After 1 month, she presented with dyspnoea and HRCT Chest revealed small areas of interstitial thickening with minimal traction bronchiectasis involving bilateral lower lobes suggestive of interstitial lung disease. The dose of oral corticosteroid was increased, and the plan was to initiate cyclophosphamide. However, the patient was lost to follow-up. Over the next 2 months, she attended multiple centres with worsening dyspnoea and was treated with intravenous antibiotics, and multiple immunosuppressants like Rituximab, tacrolimus, and plasmapheresis in view of refractory disease with type 1 respiratory failure. The bronchoalveolar lavage study was not suggestive of infection. As her disease was refractory to the above-mentioned drugs, she was initiated on tofacitinib 5mg twice a day and pulse methylprednisolone. Though she still requires domiciliary oxygen, her dyspnoea has improved and oxygen requirement has reduced.

### Case 4

A 32-year-old male presented to an outside centre with inflammatory polyarthritis, photosensitive rash, bilateral proximal muscle weakness for 4 months, and dyspnoea which progressed rapidly from MMRC grade 2 to grade 4 over 1 month. Hence, he was initiated on 2g/kg intravenous immunoglobulin and high-dose corticosteroid. As there was no clinical improvement, he was treated with intravenous cyclophosphamide (500mg fortnightly for 6 doses) and oral cyclosporine 200 mg per day. However, the patient developed candida esophagitis which required treatment with oral fluconazole and cyclosporine was stopped. 7 months into the illness, he presented to us with hypoxemia. He was amyopathic and had heliotrope rash, Shawl sign, V sign, and palmar papules (Figure 1e). HRCT Chest showed features of ILD. He was started on a combination of oral prednisolone 1mg/kg, mycophenolate mofetil, and tacrolimus. Over the next 24 months, his dyspnoea severity has reduced to grade 1 MMRC symptoms, serial pulmonary function tests showed an improving trend, and he is currently on 2gram/day of mycophenolate mofetil, 4mg/day of tacrolimus and 5mg/day oral prednisolone.

### Case 5

A 46-year-old lady presented with intermittent fever, polyarthritis, and photosensitive rash on her lower eyelids and the anterior aspect of neck for the last 2 months. She denied any shortness of breath or muscle weakness. Upon examination, the V sign, Gottron’s papules (**Figure 1F**) and synovitis of small joints of the hand were seen. With a provisional diagnosis of clinically amyopathic dermatomyositis, myositis autoantibody panel was tested which showed anti-MDA5 +++. HRCT Chest was normal. She was treated with 1mg/kg oral prednisolone following which she showed a good clinical response. Two weeks later she was admitted with steroid-induced gastritis which improved with a reduction of prednisolone dose. Two weeks after discharge from the hospital, she presented to us with progressive dyspnoea and was found to be hypoxemic. There was no history of fever or cough. HRCT Chest revealed ground-glass attenuation and septal thickening in both lower lobes, and upper lobes (**Figure 1G**).

With differentials of rapidly progressive interstitial pneumonia secondary to anti-MDA5+ Dermatomyositis versus atypical pneumonia/***pneumocystis jirovecii ***pneumonia, she was started on broad-spectrum antibiotics, oseltamivir, and cotrimoxazole, and pulsed with methylprednisolone 750mg for 3 days. The patient had progressive respiratory failure, required mechanical ventilation on day 2 of admission, and hence was started on intravenous immunoglobulin as salvage therapy. She expired on day 4 of admission due to progressive respiratory failure.

***Table 1*** shows a summary of all the five cases.

## DISCUSSION

Melanoma differentiation-associated gene 5 (MDA 5) is a cytosolic sensor of viral RNA and once it is activated, leads to a strong type 1 interferon response. Anti-MDA 5 antibody was first described by Sato et al. as an antibody targeting a 140 kD cytoplasmic polypeptide in a cohort of patients with clinically amyopathic dermatomyositis and rapidly progressive ILD.^8^ A multicentric study from France revealed 3 major phenotypes in anti-MDA 5 associated dermatomyositis 1) predominantly women with mechanic’s hand and high incidence of RP-ILD 2) predominantly arthralgia/arthritis with less incidence of RP-ILD 3) men with vasculopathy features like cutaneous ulcers, Raynaud’s phenomenon, and calcinosis. Rapidly progressive ILD was found in 17 % of the patients in this study.^3^ In a study of 76 Italian patients with dermatomyositis/polymyositis, anti MDA5 antibody was detected in 5 patients by immunoprecipitation and all 5 patients had clinically amyopathic dermatomyositis with typical dermatomyositis skin lesions. RP- ILD was found in only 1 patient.^9^ In the Pittsburgh myositis cohort, anti MDA 5 positivity was seen in 13.1% of patients, and RP- ILD was seen in 50% of patients with anti MDA 5 antibody.^10^ In a Japanese cohort of 79 patients with dermatomyositis^11^, 17 patients were positive for anti-MDA 5 autoantibody and RP ILD was seen in 12/17 patients (71%). In a multi-centre retrospective cohort study of 75 patients with myositis associated ILD, 14 patients (18.7%) were positive for anti MDA 5 antibody. The all cause 1 year mortality was higher in anti MDA 5 positive patients compared to anti MDA 5 negative group. This study also found that the 1-year all-cause mortality was higher in patients with RP-ILD and acute respiratory failure compared to patients diagnosed in the outpatient setting.^12^

There is a paucity of data from the Indian subcontinent on the clinical profile and response to therapy in anti-MDA 5 antibody-associated RP-ILD. In the largest description of anti-MDA 5 associated dermatomyositis from India comprising of 29 adult patients^13^, RP- ILD was seen in only 2 patients (6.8%). This was a retrospective study and both patients with RP-ILD succumbed to their disease. The authors have not specifically described the clinical characteristics of patients with RP-ILD in this study. In a bicentric prospective observational study, Dunga et al^14^ described 21 adult patients with anti-MDA 5 + dermatomyositis. Four patients had RP-ILD out of which 3 died due to RP-ILD. The median follow up was 2.5 months. All patients had received high-dose pulse methylprednisolone and the one patient who survived had stabilisation of lung function with high-dose pulse methylprednisolone, cyclophosphamide, 7 sessions of plasmapheresis and tacrolimus. The presence of RPILD was associated with a 16.2 times higher likelihood of mortality in this study. In a single centre cohort of 18 patients with anti MDA 5 dermatomyositis (adult-13, juvenile- 5), Devarasetti et al.^15^ found RP-ILD in 2 adult patients and both patients expired. Both patients had cutaneous ulcers, Gottron’s papule, Gottron’s sign, clinically amyopathic dermatomyositis and received agents which included glucocorticoids, cyclophosphamide, tacrolimus, plasmapheresis, Rituximab, and mycophenolate mofetil. **Table 2** shows a summary of the previously published case reports and case series describing the clinical characteristics, therapeutics and treatment outcomes of patients with anti MDA5 antibody associated RP-ILD.

**Table 2.** Summary of clinical characteristics, therapeutics, and treatment outcome of patients with anti MDA5 associated RP-ILD.

**Serial no/Reference no**	**Author**	**Year**	**Type of study**	**No. of patients**	**Age**	**Gender**	**Extra pulmonary symptoms/signs**	**Treatment**	**Outcome**
1/24	Englert B et al.	2024	Case report	2	42 30	M M	Arthralgia, psoriasiform rash, GP	Prednisolone, CsA, TOFA, CYC, IVIG	Both patients expired
2/25	Chua CG et al.	2024	Case report	1	30	M	Myalgia, fever, typical dermatomyositis rash	Pulse MP, RTX, TOFA, PLEX, IVIG, TAC, daratumumab	Improvement
3/26	Onose T et al.	2023	Case report	1	53	M	Refractory panniculitis, fever, rash	Pulse glucocorticoids, CYC, TAC, TPE	Death
4/27	Roeser et al.	2023	Case report	2	62 35	F M	Arthralgia, HR, GP, cardiomyopathy	Pulse methylprednisolone, tacrolimus, cyclophosphamide, tofacitinib, plasma exchange	Improvement
5/28	Holzer M et al.	2022	Case report	1	19	M	Fever, proximal muscle weakness, GP, HR	Pulse methylprednisolone, IVIG, cyclophosphamide, tofacitinib, mycophenolate mofetil, Rituximab, cyclosporine A, Anakinra, daratumumab	improvement
6/29	Nascimento J et al.	2022	Case report	1	49	M	Oropharyngeal ulcers, odynophagia, MH, GP, HR	Pulse MP, IVIG, CYC, RTX, MMF	Improvement
7/30	Witkowska AB et al.	2022	Case report	1	40	M	Oral ulcers, arthralgia, RP, CU	Pulse MP, CYC, TPE, RTX, MMF	Improvement
8/31	MarchisA et al.	2021	Case report	1	44	M	Fever, myalgia	Glucocorticoid, CYC, PLEX, TOFA, TAC, lung transplantation	Improvement
9/32	Mehta AA et al.	2021	Case report	2	37 42	F F	Fever, myalgia, RP, photosensitivity	Pulse MP, IVIG, CYC, RTX	Both patients expired
10/33	Chen C et al.	2021	Case report	1	38	F	Polyarthritis, GP, HR	Pulse MP, CYC, CsA	Improvement
11/34	Zhang X et al.	2021	Case series	6	51.5 (mean age)	3 M 3F	Rash, fever, polyarthritis, muscle weakness	Pulse MP, IVIG, CsA, CYC, TAC, TCZ	Improvement in five patients 1 patient lost to follow up
12/35	Hosokawa Y et al.	2021	Case report	1	66	F	GP, proximal muscle weakness	Pulse MP, CYC, CsA, TPE, TOFA	Improvement
13/36	Kunitomo Y	2020	Case report	1	62	M	HR, CU, adenocarcinoma lung	Pulse MP, CYC, TPE	Death
14/37	Saito T et al.	2020	Retrospective case series	6	56 (mean age)	4 F 2 M	GS, inverse Gottron sign, HR, MH, VS, CU, nail fold changes	Pulse MP, IVIG, CsA, CYC, TAC RTX, MMF, TPE	4 patients alive with improvement 2 patients died
15/38	Yamagata A et al.	2020	Case report	3	60 60 62	F F M	GS, inverse Gottron sign, HR, SS, VS, periungual erythema, Holster sign, fever, arthralgia	Glucocorticoid, CYC, TAC, CsA	Improvement In all three patients
16/39	Li ZY et al.	2020	Case report	1	27	F	GS	Pulse MP, IVIG, RTX.	Death
17/40	Takada T et al.	2021	Case report	1	41	F	Proximal muscle weakness, HR, GS	Pulse MP, CsA, TPE, double lung transplant	Death
18/41	Fenando A et al.	2020	Case report	1	46	F	Fever, digital hyperkeratosis, periungual erythema	Pulse MP, RTX, TAC	Worsening lung function
19/42	Huang K et al.	2020	Case series	4	61 56 53 18	F F M F	HR, GS, ulcerative rash, PP, periungual erythema, proximal muscle weakness	Pulse glucocorticoid, IVIG, CYC, RTX, Double lung transplantation	3 patients-Remission 1 patient died
20/43	So H et al.	2018	Case series	4	49 50 38 48	F M M M	Fever, weight loss, HR, VU, GS	RTX, prednisolone, TAC	Improvement in all patients
21/44	Alqatari S et al.	2018	Case report	1	49	F	Proximal muscle weakness, arthralgia, dysphagia, oral ulcers, rash, RP	Pulse MP, RTX, IVIG, CYC, TAC, PLEX	Death
22/45	Endo Y et al.	2018	Case report	1	71	F	Proximal muscle weakness, HR, GS, inverse Gottron sign	Pulse MP, CYC, CsA, TPE	Improvement
23/46	Leclair V et al.	2018	Case report	1	38	F	Proximal muscle weakness, HR, GP, periungual erythema, MH	Pulse MP, CYC, double lung transplantation	Improvement
24/47	De Backer E et al.	2018	Case report	1	55	M	GP, MH, arthritis, fever, muscle weakness	Pulse MP, CYC	Death
25/48	Osawa T et al.	2018	Case report	1	61	F	GP, MH, HR	Pulse MP, CYC, CsA, TPE	Death
26/49	Hoa S et al.	2017	Case series	9	52 (mean age)	4 F 5 M	HR, GS, VS, SS, PP, CU, panniculitis, MH, fever, arthritis, proximal muscle weakness	Pulse MP, IVIG, CsA, CYC, TAC, RTX, MMF, TPE	3 deaths, 6 patients survived
27/50	Nandy A et al.	2018	Case report	1	44	F	Fever, oral aphthae, GP	Pulse MP, CYC	Death
28/51	Hisanaga J et al.	2017	Case report	1	57	F	HR, GS, PP, CU	Pulse MP, IVIG, CsA, CYC, RTX, MMF, TPE	Improvement
29/52	Tokunaga K et al.	2017	Case report	2	71 69	F F	Fever, VS Polyarthralgia, GS	Pulse MP, TAC, CYC, CsA, RTX, TCZ	Both patients died
30/53	Górka J et al.	2015	Case report	1	59	M	Fever, arthritis, GS, PP, MH	High dose corticosteroid	Death
31/54	Silveira MG et al.	2016	Case report	1	42	M	Fever, MH, GP	Pulse MP, TAC, polymyxin B hemoperfusion, PLEX, IVIG	Improvement
32/55	Koichi Y et al.	2017	Case report	1	71	F	HR, PP, CU	Pulse MP, TAC, CYC, polymyxin B hemoperfusion, IVIG, RTX	Improvement
33/56	Zou J et al.	2014	Case report	4	56 46 54 51	F F F M	HR, GP, CU	Prednisolone, CsA, IVIG, Basiliximab	3 patients Improved 1 patient died
34/57	Teruya A et al.	2013	Case report	1	52	M	GP, fever	Pulse MP, CsA, polymyxin B hemoperfusion	Improvement
35/58	Chong KM et al.	2023	Retrospective case series	4	60 72 48 61	F F M F	Fever, arthralgia, GP, MH, HR, SS, CU, ragged cuticles	Pulse MP, CYC, TOFA, RTX	3 alive 1 died
36/59	Lenna L et al.	2023	Retrospective case series	6	67 30 55 61 60 58	M M M F M M	Articular, cutaneous	Pulse MP, CYC, TAC, PLEX	4 died, 2 improved

GP: Gottron’s papule; GS: Gottron’s sign; HR: heliotrope rash; MH: Mechanic’s hands; CU: cutaneous ulcers; VS: v sign; SS: shawl sign; VR: vasculitic rash; RP: Raynaud’s phenomenon; PP: palmar papule; MP: Methyl prednisolone; MMF: Mycophenolate; CsA: Cyclosporine A; CYC: Cyclophosphamide; RTX: Rituximab; TAC: Tacrolimus; TOFA: Tofacitinib; TCZ: Tocilizumab; PLEX: Plasmapheresis; IVIG: intravenous immunoglobulin, TPE: Therapeutic plasma exchange.

In our cohort of seven patients with anti MDA 5 positive dermatomyositis, RP-ILD was seen in 5 patients (71.4 %) which is much higher than the previously published literature from India.^13,14,15^ Three patients (Case 3, Case 4, Case 5) had dermatomyositis specific skin lesions. Though Case 1 and Case 2 did not have dermatomyositis specific skin lesions, anti MDA5 related RP-ILD without skin findings of dermatomyositis has been previously reported.^16,17^ This highlights the fact that a myositis panel should be tested in all patients with idiopathic ILD and not just those patients with dermatomyositis.^18,19^ Polyarthritis was seen in all patients with RP-ILD in our cohort (100%) and as it preceded the development of respiratory symptoms by months, these patients might be misdiagnosed as seronegative Rheumatoid arthritis especially in the background of a negative Rheumatoid factor, anti CCP and ANA. All the patients had amyopathic presentation with normal creatinine kinase levels. ANA by immunofluorescence was negative in all the 5 patients (100%). The two patients who died had elevated serum lactate dehydrogenase and ferritin levels. High serum LDH and ferritin levels have been previously described as predictors of mortality in anti MDA 5 associated RP-ILD.^20,21^ Though prior literature shows increased prevalence of anti-Ro 52 in anti MDA 5+ DM and dual positivity to be associated with RP-ILD and poor prognosis of RP-ILD^22^, we had only 1 patient with dual positivity (Case 3). As prior literature suggests improved survival with combination of immunosuppressants/immunomodulators in treatment of anti MDA 5 related RP-ILD^22,23^, we followed combination therapy in all our patients. The fact that three of our patients have survived and is showing improving/stabilisation of lung function is reassuring that once patients survive the period of hospitalisation with RP-ILD, they may show signs of clinical improvement.

The small number of RP-ILD cases and single centre setting are the limitations of our case series. The follow up data is available for only three patients which is another limitation of the present study. As the study was done in a tertiary care referral centre, it is possible that we have encountered the more severe manifestations associated with anti MDA5 antibody and this might have contributed to the high frequency of RP-ILD cases in our cohort. The strengths of our study include the longitudinal design and long duration of follow-up with regular monitoring of all patients who survived.

## CONCLUSION

To conclude, we found a high incidence of RP-ILD in our patients with anti MDA5 positive DM. A clinically amyopathic presentation with polyarthritis and negative ANA defined the clinico-serological profile of our anti- MDA 5 related RP-ILD patients. The survivors in our study received a non-uniform combination of immunosuppressants highlighting the importance of choosing a therapeutic regime on a case-to-case basis in the absence of robust data to guide therapy. Larger studies involving patients from multiple ethnicities is required to accurately determine the phenotype and long-term treatment outcomes of RP-ILD patients.
